# The Unique Structure of the Apicoplast Genome of the Rodent Malaria Parasite *Plasmodium chabaudi chabaudi*

**DOI:** 10.1371/journal.pone.0061778

**Published:** 2013-04-16

**Authors:** Shigeharu Sato, Abdul K. Sesay, Anthony A. Holder

**Affiliations:** 1 Division of Parasitology, MRC National Institute for Medical Research, London, United Kingdom; 2 Genomics Facility, Division of Systems Biology, MRC National Institute for Medical Research, London, United Kingdom; Université Pierre et Marie Curie, France

## Abstract

The apicoplast, a non-photosynthetic plastid of apicomplexan species, has an extremely reduced but highly conserved genome. Here, the apicoplast genome of the rodent malaria parasite *Plasmodium chabaudi chabaudi* (Pcc) isolate CB was characterized. Although the set of genes in the genome is identical, the copy number of some tRNA genes differs between Pcc and other *Plasmodium* species because the Pcc DNA has only one rRNA/tRNA gene cluster, which is normally duplicated in other species. The location of the duplicated *trnR(ACG)* and *trnM* implies that one of the duplicated clusters in the ancestral molecule has been lost due to an intramolecular recombination event. The Pcc DNA occurs in two isoforms with an internal inversion between them. The presence of a unique variant in the duplicated *trnT* gene suggests that the two isoforms are interconvertible. This is the first report of the complete nucleotide sequence of a *Plasmodium* apicoplast DNA.

## Introduction

The apicoplast is a secondary plastid of parasites belonging to the phylum Apicomplexa [1]–[3]. Although it lacks photosynthetic activity, the organelle is critical for survival and growth [4]. Like other non-photosynthetic plastids [5], the apicoplast has a much smaller genome than those of photosynthetic eukaryotes. The apicoplast genome first analyzed was that of the human malaria parasite *Plasmodium falciparum* (Pf), which is encoded in a circular DNA of 35 kb in size [6]. The Pf apicoplast genome encodes large and small subunit ribosomal RNAs and 25 different tRNA species. The genes of two rRNAs (*rrl* and *rrs*) are arranged head-to-head and form a cluster with nine tRNA genes. The cluster is duplicated to form an inverted repeat (IR). Proteins encoded by this tiny genome are mostly involved in either transcription or translation and their genes comprise two unidirectional clusters in the single copy region following the *trnT* gene at one end of each IR unit.

The Pf plastid DNA sequence reported by Wilson and colleagues was incomplete as it lacked a short sequence between the two IR units on the circular DNA [6]. Recently, Arisue and colleagues analyzed the corresponding part of apicoplast genomes of eight *Plasmodium* species other than Pf [7]. The results revealed that the gene repertoire, gene arrangement, and other structural attributes are strongly conserved in those species, except for a peculiarity in the plastid DNA of the rodent malaria parasite *P. chabaudi chabaudi* (Pcc) isolate AS. *Plasmodium* species other than Pcc had a gene specifying tRNA-Arg(ACG) (*trnR(ACG)*) between the genes for tRNA-Met (*trnM*) and tRNA-Val (*trnV*) in each IR unit. By contrast, the sequence between *trnM* and *trnV* in Pcc AS DNA was significantly divergent. An unusual *trnR(ACG)* (trnR(ACG)*) was predicted in this region, but the product of this putative gene cannot be tRNA-Arg(ACG) because the RNA is unlikely to have ACG as the anticodon.

The missing tRNA-Arg(ACG) is essential for decoding CGN codons that frequently occur in the Pcc plastid genome (Table 1). Consequently, unless the apicoplast imports the tRNA from the cytosol, the organellar genome should contain *trnR(ACG)*. To clarify this, we analyzed the whole genomic DNA of Pcc isolate CB by high throughput sequencing (HTS). In conjunction with data obtained with Sanger sequencing, the HTS data suggest that the plastid genome of Pcc is unique in the apicoplast DNAs of *Plasmodium* spp.

**Table 1:** Codon usage in the plastid genome of *Plasmodium chabaudi chabaudi* isolates and *Plasmodium falciparum*.

			Number of codons a			
Amino acid	1 letter code	Codon	P.c.c. AS b	P. c. c. CB c	P. f. C10 d	Decoding tRNA e
– f	–	TAA	23	22	24	–
–	–	TAG	0	1	0	–
–	–	TGA	8	8	7	–
Ala	A	GCA	21	21	16	tRNA-Ala(ACC)
Ala	A	GCC	1	1	1	tRNA-Ala(ACC)
Ala	A	GCG	4	4	0	tRNA-Ala(ACC)
Ala	A	GCT	37	37	41	tRNA-Ala(ACC)
Cys	C	TGC	2	5	1	tRNA-Cys(GCA)
Cys	C	TGT	84	81	82	tRNA-Cys(GCA)
Asp	D	GAC	11	10	3	tRNA-Asp(GUC)
Asp	D	GAT	126	125	127	tRNA-Asp(GUC)
Glu	E	GAA	134	134	145	tRNA-Glu(UUC)
Glu	E	GAG	11	11	8	tRNA-Glu(UUC)
Phe	F	TTC	4	4	5	tRNA-Phe(GAA)
Phe	F	TTT	429	428	457	tRNA-Phe(GAA)
Gly	G	GGA	83	84	85	tRNA-Gly(UCC)
Gly	G	GGC	0	0	0	tRNA-Gly(ACC)
Gly	G	GGG	17	16	13	tRNA-Gly(UCC)
Gly	G	GGT	116	116	120	tRNA-Gly(ACC)
His	H	CAC	3	3	0	tRNA-His(GUG)
His	H	CAT	58	58	70	tRNA-His(GUG)
Ile	I	ATA	662	660	758	tRNA-Ile(GAU)
Ile	I	ATC	8	9	8	tRNA-Ile(GAU) g
Ile	I	ATT	584	585	520	tRNA-Ile(GAU)
Lys	K	AAA	888	883	916	tRNA-Lys(UUU)
Lys	K	AAG	47	48	53	tRNA-Lys(UUU)
Leu	L	CTA	7	6	11	tRNA-Leu(UAG)
Leu	L	CTC	0	0	0	tRNA-Leu(UAG)
Leu	L	CTG	0	0	0	tRNA-Leu(UAG)
Leu	L	CTT	9	9	4	tRNA-Leu(UAG)
Leu	L	TTA	898	900	886	tRNA-Leu(UAA)
Leu	L	TTG	31	29	19	tRNA-Leu(UAA)
Met (initial)	M	ATG	31	31	31	tRNA-fMet(CAU)
Met (internal)	M	ATG	47	46	55	tRNA-Met(CAU)
Asn	N	AAC	20	21	7	tRNA-Asn(GUU)
Asn	N	AAT	1071	1066	1116	tRNA-Asn(GUU)
Pro	P	CCA	26	26	27	tRNA-Pro(UGG)
Pro	P	CCC	1	1	1	tRNA-Pro(UGG)
Pro	P	CCG	0	0	1	tRNA-Pro(UGG)
Pro	P	CCT	77	77	76	tRNA-Pro(UGG)
Gln	Q	CAA	113	113	118	tRNA-Gln(UUG)
Gln	Q	CAG	6	6	9	tRNA-Gln(UUG)
Arg	R	AGA	91	91	85	tRNA-Arg(UCU)
Arg	R	AGG	3	3	3	tRNA-Arg(UCU)
Arg	R	CGA	0	0	0	tRNA-Arg(ACG)
Arg	R	CGC	0	0	0	tRNA-Arg(ACG)
Arg	R	CGG	0	0	0	tRNA-Arg(ACG)
Arg	R	CGT	12	12	14	tRNA-Arg(ACG)
Ser	S	AGC	5	5	0	tRNA-Ser(GCU)
Ser	S	AGT	107	107	107	tRNA-Ser(GCU)
Ser	S	TCA	103	102	98	tRNA-Ser(UGA)
Ser	S	TCC	7	7	4	tRNA-Ser(UGA)
Ser	S	TCG	1	2	3	tRNA-Ser(UGA)
Ser	S	TCT	140	140	132	tRNA-Ser(UGA)
Thr	T	ACA	111	111	101	tRNA-Thr(UGU)
Thr	T	ACC	0	0	1	tRNA-Thr(UGU)
Thr	T	ACG	3	2	2	tRNA-Thr(UGU)
Thr	T	ACT	109	109	106	tRNA-Thr(UGU)
Val	V	GTA	79	78	79	tRNA-Val(UAC)
Val	V	GTC	1	1	0	tRNA-Val(UAC)
Val	V	GTG	7	7	1	tRNA-Val(UAC)
Val	V	GTT	68	68	48	tRNA-Val(UAC)
Trp	W	TGG	30	30	29	tRNA-Trp(CCA)
Tyr	Y	TAC	9	9	8	tRNA-Tyr(GUA)
Tyr	Y	TAT	807	806	777	tRNA-Tyr(GUA)
		Sum	7391	7375	7419	
		Total nt	22173	22125	22257	

a The sum for each codon species present in all the protein coding sequences annotated in the apicoplast DNA.

b AB649423.1 [7].

c HF563595/HF563596 (this work).

d X95275.2/X95276.2 [6].

e Proposed tRNA species decoding the codon [10].

f Termination codon.

g It is not certain if tRNA-Ile(GAU) can decode ATC codons to Ile.

## Results and Discussion

The whole genomic DNA of Pcc isolate CB was analyzed by high throughput sequencing (HTS) on the Illumina platform. Obtained reads covered the entire length of the previously partly sequenced plastid DNA of Pcc isolate AS [7] at an average depth of 50× without gaps (Table 2 and Figure S1A). The consensus sequence of HST reads had 42 differences from the 29,198 nt AS reference sequence (Table 3). Most of this variation within protein coding sequences was synonymous base substitution, but five SNPs and three deletions affect the amino acid sequences encoded by *rpl23, rpl4*, *rps5*, *rpoC1*, *rpoC2A*, *rpoC2B* and *ORF91*. Variations located within *trnT* or in intergenic regions surrounding *trnM* were not unique at each position, implying that these parts are duplicated in the Pcc plastid DNA.

**Table 2:** Alignment of HTS reads of *Plasmodium chabaudi chabaudi* isolate CB on available sequence data of *P. c. c.* isolate AS and a mouse single-copy gene.

Kind	Reference sequence	Length (bp)	Read count	Average coverage (x)	Ratio a
*P. c. c.* AS nuclear chromosomal/mitochondrial contigs b					
	chab01	581723	140320	23.88	1.00
	chab02	498758	121750	24.18	1.01
	chab03	624158	145688	23.07	0.97
	chab04	794008	182819	22.78	0.95
	chab05	935796	227947	24.11	1.01
	chab06	916956	218714	23.60	0.99
	chab07	1169068	285453	24.16	1.01
	chab08	1360204	329661	24.00	1.00
	chab09	1345096	328190	24.16	1.01
	chab10	1634725	397118	24.07	1.01
	chab11	1720574	415441	23.92	1.00
	chab12	1682238	411364	24.22	1.01
	chab13	2616835	632816	23.95	1.00
	chab14	2514833	615891	24.27	1.02
	PCHAS_MIT	5949	12531	209.02	8.75
*P. c. c.* AS plastid DNA					
	AB649423 c	29198	14949	50.97	2.13
Mouse single-copy gene					
	Cela2a d	11041	32	0.28	0.01

a Ratio of the average coverage to the average of 14 chromosomal contigs (chab01-14).

b These preliminary genomic contigs (dated December 2012) were produced by the Wellcome Trust Sanger Institute and can be obtained from ftp://ftp.sanger.ac.uk/pub/pathogens/Plasmodium/chabaudi.

c *Plasmodium chabaudi* apicoplast DNA, partial sequence [7].

d The genomic locus specifying chymotrypsin-like elastase family, member 2A. GRCm38.p1 C57BL/6J Chromosome: 4; NC_000070.6 (141814963.141826003, complement).

**Table 3:** Variations identified in the plastid DNA sequence between *Plasmodium chabaudi chabaudi* isolates CB and AS.

Reference a		CB Consensus		Reads			Gene		
Position	Residue b	Residue b	Type c	Coverage	Counts	Frequencies (%)	Name d	Nucleotide e	Amino acid f
28	C	C/T	(SNV)	20	18/2	90.0/10.0	trnT(UGU)	(C28T)	–
2121	A	T	SNV	54	54	100.0	rpl4	A324T	(Ile108; synomymous)
2206	T	A	SNV	36	35	97.2	rpl4	T409A	Tyr137Asn
2256	T	C	SNV	38	37	97.4	rpl4	T459C	(Asn153; synomymous)
2584	C	A	SNV	44	44	100.0	rpl23	C180A	Asp60Glu
3039	A	G	SNV	29	29	100.0	rpl2	A423G	(Ser141; synomymous)
3751	C	T	SNV	38	38	100.0	rps3	C97T	(L33; synomymous)
5516	C	T	SNV	27	27	100.0	rps8	C225T	(Asn75; synomymous)
6535.6537	AAT	–	Deletion	43	43	100.0	rps5	AAT(301.303) deletion	Asn101 deletion
6741	T	C	SNV	51	51	100.0	rps5	T507C	(Tyr169; synomymous)
6822	T	C	SNV	47	47	100.0	rps5	T588C	(Cys196; synomymous)
7209	A	–	Deletion	56	55	98.2	ORF91	A241 deletion	Ile81-Met92 deletion
8829	T	C	SNV	55	55	100.0	tufA	T246C	(Cys82;synomymous)
8835	G	A	SNV	55	55	100.0	tufA	G252A	(Gly84; synomymous)
(10214)	–	T	Insertion	54	54	100.0	trnG(ACC)	TT(11.12)TTT	–
10489	G	A	SNV	46	46	100.0	ORF129	G108A	(L36; synomymous)
11639	T	C	SNV	51	51	100.0	clpC	T876C	(Ile292; synomymous)
12566	T	C	SNV	59	59	100.0	clpC	T1803C	(Cys601; synomymous)
12932	C	T	SNV	53	53	100.0	clpC	C2169T	(Asn723; synomymous)
14009	T	C	SNV	56	56	100.0	rps2	A474G	(Lys158;synomymous)
15593	G	A	SNV	38	38	100.0	rpoC2B	C183T	(Tyr61; synomymous)
15692	C	A	SNV	28	28	100.0	rpoC2B	G84T	Leu28Phe
15719.15727	TATTTTATT	–	Deletion	26	26	100.0	rpoC2B	AATAAAATA(49.57) deletion	Asn17-Ile19 deletion
16721	C	T	SNV	62	62	100.0	rpoC2A	G673A	Val225Ile
16908	A	G	SNV	40	40	100.0	rpoC2A	T486C	(Asn162;synomymous)
17277	A	G	SNV	48	47	97.9	rpoC2A	T117C	(Phe39; synomymous)
17534	T	A	SNV	48	48	100.0	rpoC1	A1064T	Asn535Ile
18892	A	G	SNV	40	40	100.0	rpoC1	T246C	(Asn82; synomymous)
22427	A	G	SNV	32	32	100.0	ORF101	T84C	(Tyr13; synomymous)
24166	G	A/G	(SNV)	99	56/43	56.6/43.4	trnT(UGU)	(C28T)	–
26918	T	T/G	(SNV)	58	41/17	70.1/29.3	(trnM>rrl)	–	–
26920	T	T/−	(Deletion)	64	46/18	71.9/28.1	(trnM>rrl)	–	–
26928	A	A/T	(SNV)	61	43/18	70.5/29.5	(trnM>rrl)	–	–
26931	T	T/A	(SNV)	63	45/18	71.4/28.6	(trnM>rrl)	–	–
27032	A	A/G	(SNV)	72	55/17	76.4/23.6	(trnM<>trnV)	–	–
27279	A	G	SNV	41	39	95.1	trnL(UAG)	A26G	–
27873	T	–	Deletion	63	63	100.0	rrs	T314 deletion	–
28345	T	A	SNV	42	42	100.0	rrs	T786A	–
28346	A	T	SNV	42	42	100.0	rrs	A787T	–
28347	A	T	SNV	41	41	100.0	rrs	A788T	–
28348	T	A	SNV	39	39	100.0	rrs	T789A	–
29013	T	C	SNV	47	47	100.0	(rrs>trnI)	–	–

a AB649423.1 [7].

b –: gap. For variation that was not unique, the two most major variations are given at each side of/.

c SNV: single nucleotide variation. Variation that was not unique is shown in parenthesis.

d Name of the gene containing each variation. For variation in the non-coding region, nearest genes are shown in parenthesis.

e Position in the coding sequence. Position is that in the AS sequence, and those which were not unique are given in parenthesis. –: a variation in a non-coding region.

f Change in the amino acid sequence of the CB version caused by each variation. Position is that in the AS sequence. Synonymous variations are shown in parenthesis. –: a variation outside a protein coding sequence.

We searched for reads encoding a regular tRNA-R(ACG) in our HTS data using the *trnR(ACG)* sequence of *P. berghei* (Pb), another rodent malaria parasite [7] as the reference (Figure S1B). The result clearly suggested that a previously unidentified *trnR(ACG)* exists in the Pcc CB DNA along with the peculiar trnR(ACG)* which Arisue et al. had found. Our data also suggested that both the regular and peculiar *trnR(ACG)* genes precede *trnM*, and share an identical intergenic sequence.

To further examine this finding, we next searched for HTS reads containing the *trnM* sequence (Figure S1C). The reads we identified were classified into two groups. The consensus of one group perfectly matched the reference sequence containing *trnM* (*trnM-1*). On the other hand, the consensus of the other group had several conflicts with the reference sequence outside the tRNA gene (*trnM-2*), despite the fact that this gene encodes an identical tRNA-Met. As suggested by our earlier search, the 3′ end of the regular *trnR(ACG)* appeared in front of *trnM-2*. These results confirmed that the Pcc plastid DNA has two distinct *trnM* genes specifying an identical tRNA-Met, and that the newly found *trnM-2* adjoins the missing *trnR(ACG)*.

**Figure 1:**
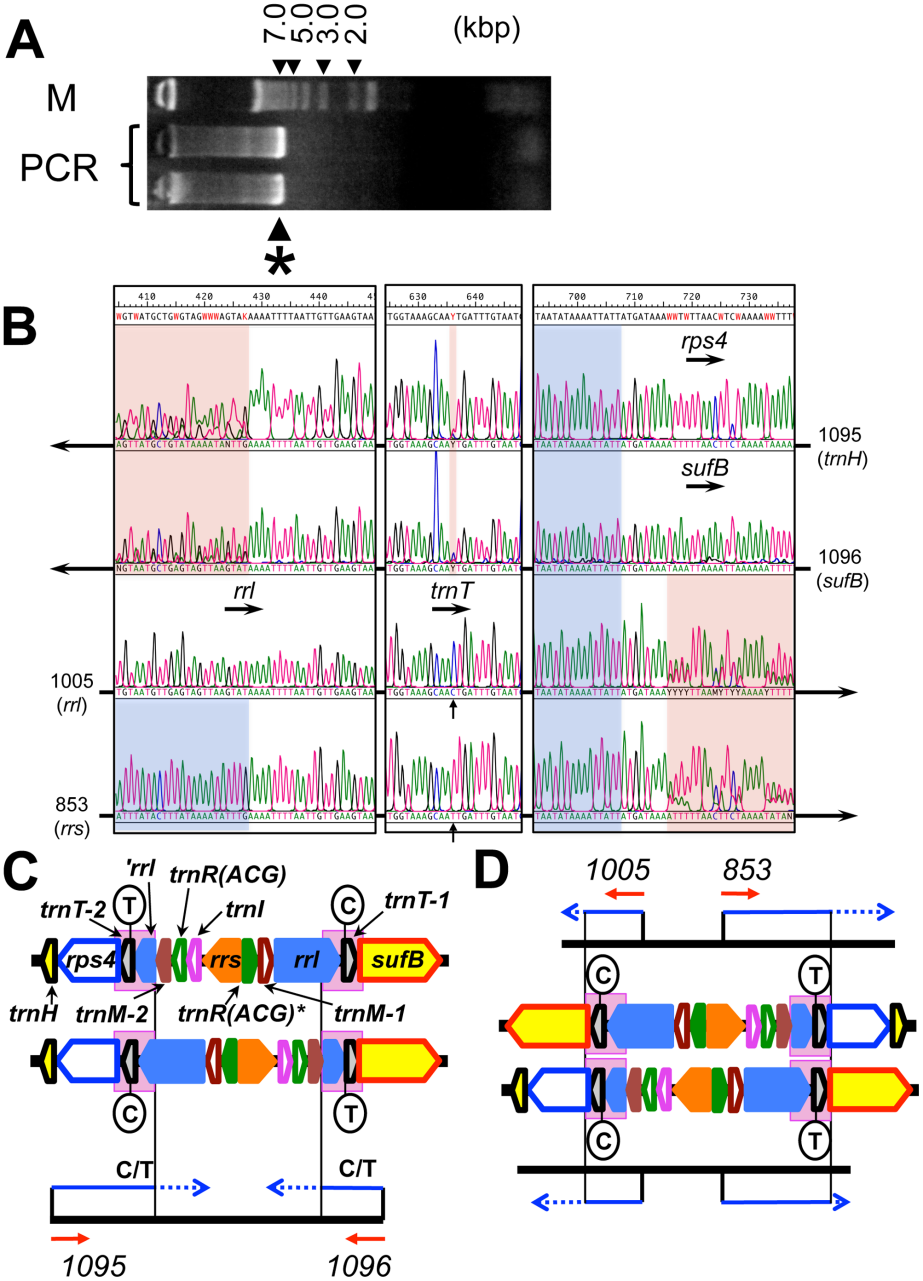
The rRNA/tRNA gene cluster of *Plasmodium chabaudi chabaudi* CB plastid DNA. (A) Part of the Pcc CB plastid DNA was amplified by PCR from total parasite DNA with the primers 1095 and 1096 (see Table 4 for details of each primer). The product was fractionated on an agarose gel along with DNA size markers (M). DNA that appeared as a single band (*) was collected and analyzed further. (B) Alignment of trace data obtained for the 6 kb PCR product by PCR-direct sequencing with primers that anneal at an end (1095, 1096) or an internal position (1005, 853). Three parts of the alignment are presented in boxes with arrows representing the direction of the sequencing reaction starting from each primer; the name of an encoded gene is given with a horizontal arrow representing the direction of transcription. Non-coding sequence and ambiguous sequencing data due to the presence of two different sequences are highlighted with blue and red, respectively. Because *rps4* and *sufB* share an identical sequence from the 1st to the 8th nucleotide of their coding sequence, the highlighted region in red starts at the 9th residue in the sequencing data from 1005 and 853. The C/T transition at position 28 of *trnT*, which is clearly identifiable in the sequencing data analyzed from inside (1005/853) but not in those from outside (1095/1096), is indicated with a vertical arrow. (C and D) Schematic representation of the PCR product. Selected genes in the region including the gene of the unusual tRNA-Arg(ACG) (trnR(ACG)*) and the 5' truncated *rrl* (*'rrl*) are indicated with color-coded thick arrows. The PCR product (horizontal thick black bar) amplified with primers 1095 and 1096 (red arrows) was a mixture of two DNA species. Because of the coexistence of two different types of molecule, the quality of sequencing data obtained (blue arrows) abruptly dropped at the end of the short IR sequence (highlighted with pink background) and gave a mixture of two sequences (dotted arrow). The sequence of two *trnT* genes (*trnT*-1 and -2) of Pcc CB is almost the same except for the variation at their 28th residue (T and C; circled). Each *trnT* is linked with its upstream gene but not with its downstream gene. Therefore the 28th residue of the gene was a mixture of C and T when the PCR product was sequenced from the outside toward the inside (C), but the residue was uniquely identified as either C or T when the same sample was sequenced from inside to outside (D). The length of each gene is not to scale with the others in this figure.

The cluster of *trnR(ACG)* and *trnM-2* was likely present in a region that escaped analysis by Arisue et al. Accordingly, to complete the partially determined sequence of the Pcc plastid DNA, we amplified the part between *trnH* and *sufB* by PCR, obtaining a product that appeared as a 6 kb band on electrophoresis in an agarose gel (Figure 1A). Although the rate should be low, PCR amplification may result in random introduction of base substitutions into the product. In order to mitigate any risk of introducing such errors into the product for sequence analysis, we avoided subcloning the PCR fragment and determined its nucleotide sequence directly. The sequencing data from each end of the DNA suggested that both *trnH* and *sufB* are preceded by the same sequence that encodes *trnT* and *rrl* (Figure 1B). The quality of the sequencing data from each side dropped abruptly beyond the 168th nucleotide from the predicted 3′ end of *rrl*. Further analysis revealed this was due to the co-occurrence of two different sequences beyond that point.

To confirm this finding, we sequenced the same PCR product from the inside outwards toward each end. As expected, *trnT* was identified directly after *rrl* in the sequence determined from *rrl*. On the other hand, the sequence from *rrs* contained *trnI* followed by the cluster of *trnR(ACG)* and *trnM-2*. After *trnM-2*, there was a 168 nucleotide 3′-end sequence of *rrl* followed by *trnT*. The sequencing data from both *rrs* and *rrl* were identical from the beginning of the *rrl* sequence except for one nucleotide at the 28th position of *trnT* (see below), and each sequence turned into a mixture of two sequences beyond the eighth residues of *rps4*/*sufB*. These results suggest that the PCR product was a mixture of two fragments that have the same internal sequence in the opposite direction between *rps4* and *sufB* (Figure 1C and D). Hence Pcc plastid DNA does have an IR, but unlike other *Plasmodium* spp., each unit of the IR is extremely short – only 288 bp between the 168th positions from the 3′ end of *rrl* (*'rrl*) and the eighth positions of *rps4*/*sufB*.

**Figure 2:**
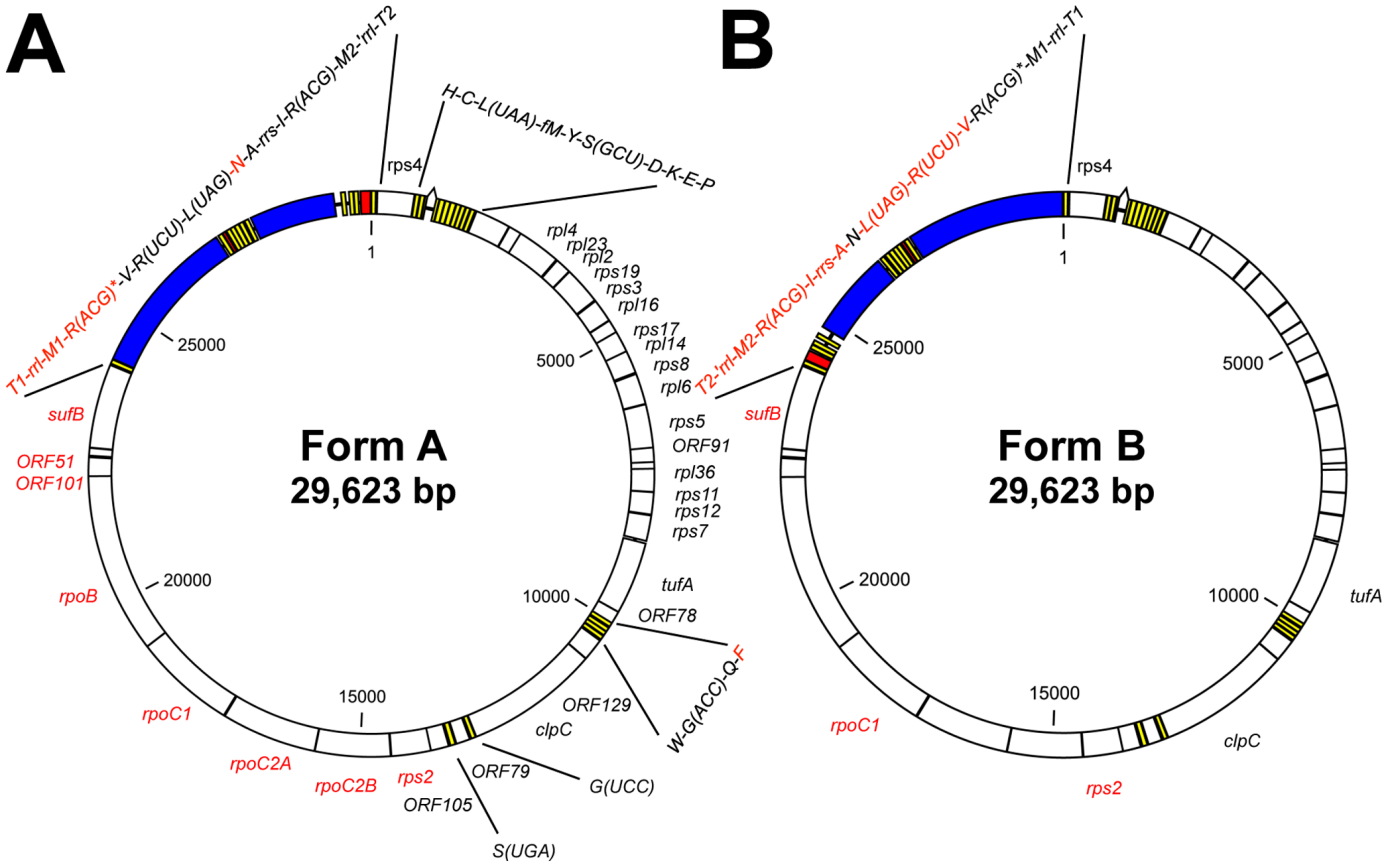
Unique forms of the plastid DNA of *Plasmodium chabaudi chabaudi* CB and their proposed origin. Schematic diagram of the two forms of the plastid DNA of Pcc CB. Each gene is represented with a box color-coded in white (one specifying a protein), blue (rRNA gene), yellow (tRNA gene) or red (a pseudogene). The colour of the name of each gene represents the transcription direction (black, clockwise; red, counter-clockwise). The bulge connecting two parts of the *trnL*(UAA) coding sequence indicates the intron. The nucleotide sequences of the complete Pcc CB plastid DNA in forms A (A) and B (B) were deposited to the DDBJ/EMBL/GenBank databases with the accession numbers HF563595 and HF563596, respectively.

In summary, using PCR to amplify the region between *rps4* and *sufB* we obtained a mixture of two DNA fragments in each of which *rrs*, *rrl* and several tRNA genes appear in the opposite direction. This indicates there are two molecular forms of Pcc CB plastid DNA. Each form has the same RNA/tRNA gene cluster only once, in contrast to all other *Plasmodium* species where it is duplicated. The direction of the RNA/tRNA gene cluster is unique to each form, and we tentatively name the DNA molecule in which *rrs* is encoded on the same strand as *rps4* as form A, whereas the other form which has *rrs* on the other strand to *rps4* as form B (Figure 2).

Curiously, we found a one-nucleotide difference between the two units of the short IR of Pcc CB. The difference, a C/T transition at the 28th position of *trnT*, probably emerged after the separation of the Pcc isolates CB and AS, because the corresponding variation was not found in *trnT* of Pcc AS [7]. Direct sequencing data for the PCR product unambiguously showed that this variation is tightly linked with the upstream gene (*rrl* or '*rrl*). By contrast, the nature of the residue at the site seemed independent of the downstream sequence (Figure 1). These observations imply that the variation in *trnT* emerged in either of the two forms only once and spread to the other form due to an intramolecular recombination event that occurred between the shorter sequences bound by the variable site and the eighth residue of *rps4*/*sufB*.

Our detailed analysis has confirmed that the Pcc plastid genome encodes the same set of tRNA species as other *Plasmodium* species. But, as the IR in the DNA molecule is exceptionally short, the copy number of each tRNA gene is distinct from that of other *Plasmodium*. The library for HTS analysis was prepared with a protocol that omits PCR amplification [8], therefore the number of reads matching each point of the reference is expected to reflect the relative abundance of the template in the starting material. The number of reads with the characteristic variation suggested that the ratio of the trnR(ACG)*-*trnM-1* and the *trnR(ACG)*-*trnM-2* gene clusters was 1∶1 (Figure S1D). This indicates that each tRNA gene is present at the same copy number in the Pcc apicoplast genome. In addition, counting the reads having each variant within those matching the *trnT* sequence (Figure S1E), we estimate the ratio between the two *trnT* genes as 1∶1.

**Figure 3:**
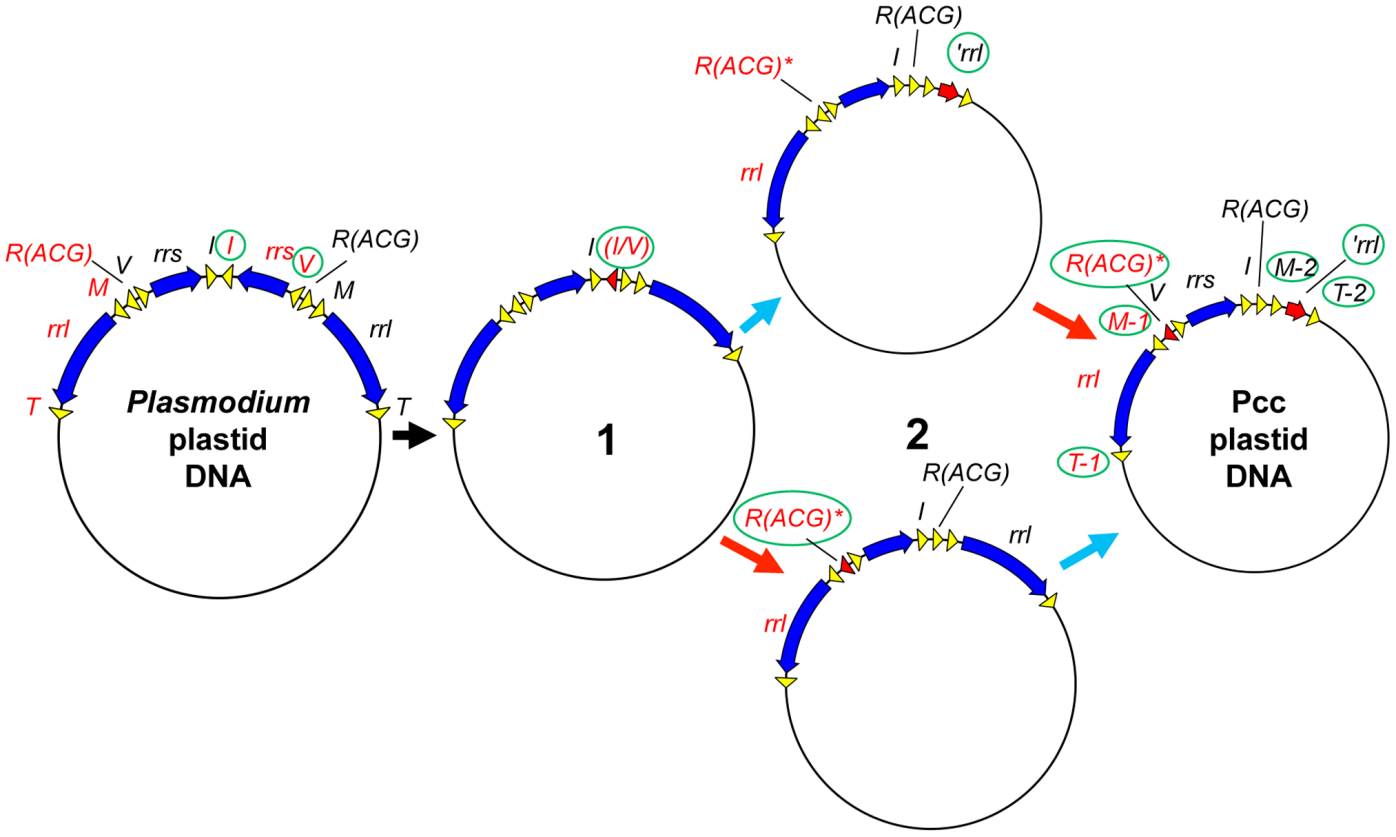
Proposed origin of the unique Pcc CB plastid DNA. An intramolecular recombination event probably between *trnI* and *trnV* caused partial deletion of one of the two IR units in the ancestral DNA molecule (thick black arrow). The chimeric *trnI*/*trnV* in the intermediate molecule (1) was lost probably because it was a pseudogene. Subsequent truncation of the 5' region of *rrl* in the affected IR unit (thick blue arrow) and degradation of the *trnR(ACG)* in the other IR unit (thick red arrow) results in the Pcc plastid DNA. The order of these two events as well as which intermediate molecule (2) was generated in this process, are unknown. Finally, differentiation between *trnM-1* and *-2* as well as between *trnT-1* and *-2* occurred and these resulted in the Pcc CB plastid DNA. Switching between forms A and B could have happened at any point of this process, and may happen frequently as suggested by the fact that the apparent molar ratio between the two forms is 1∶1. Genes are color-coded as in panels A and B, and those changing/changed are indicated with a green circle.

Consequently, the only duplicate tRNA genes that encode (practically) the same tRNA species in the Pcc apicoplast genome are *trnM* and *trnT*; generally, seven other tRNA genes (*trnA*, *trnI*, *trnL(UAG)*, *trnN*, *trnR(ACG)*, *trnR(UCU)*, and *trnV*) are also duplicated in the apicoplast genome of other species of *Plasmodium* [6], [7]. The presence of *trnI* directly upstream the *trnR(ACG)*-*trnM-2* cluster suggests that the uniqueness of the tRNA gene copy numbers in the Pcc genome was caused by an intramolecular recombination event, probably between *trnV,* which precedes *trnR(ACG)* in the IR unit of *Plasmodium* plastid DNA, and *trnI* (Figure 3). The resultant DNA molecule would have had only one copy of *rrs* in front of the remaining single-copy *trnI.* Subsequent truncation of the 5' region of *rrl* in the affected IR unit and degradation of the *trnR(ACG)* to trnR(ACG)* in the other IR unit would have given rise to the present forms of the Pcc plastid DNA.

The ratio between the two forms of Pcc plastid DNA is difficult to determine directly, but the coverage of reference sequences by the HTS reads provided an estimate. The ratio between the average coverage of a nuclear chromosome and the plastid DNA was about 1∶2 (Table 2). This suggests that the average copy number of the plastid DNA in an apicoplast is 2, given that prior to schizogony the parasite cell has only one nucleus and one apicoplast, and each nucleus contains only one haploid set of chromosomes. If this estimate is correct, one molecule each of the two forms of DNA is likely co-present with the other in the organelle. How the two forms can co-exist keeping their ratio at 1∶1 is unknown. One attractive possibility is that both forms are concatenated into a heterodimeric molecule at some point of the cell cycle (Figure S2), although there is as yet no experimental evidence supporting such a form’s physical existence in the Pcc plastid. It is also possible that there are two types of parasites in the population each with exclusively one or other form of plastid DNA molecule. Whichever is the case, it is noteworthy that the Pf apicoplast at the ring-stage seems to contain only 1 or few copies of the plastid DNA [9].

## Conclusions

We conclude that, unlike other *Plasmodium* spp., the Pcc plastid DNA has only one copy of the rRNA/tRNA gene cluster. The DNA is present in two different forms A and B that share identical sequence except for the opposite direction of the rRNA/tRNA gene cluster between *rps4* and *sufB*. The estimated ratio between forms A and B is 1∶1 and the two forms seem to be interchangeable via an intramolecular recombination in the small region between the 28th nucleotide of *trnT* and the eighth nucleotide of *rps4*/*sufB*. The coincidental presence of two *trnT*s with a C/T transition at the 28th position in the Pcc CB plastid DNA molecule suggests either that intramolecular recombination occurs frequently or that recombination events are very rare and the variants have been fixed since one event generated the two forms. Whichever is the case, the plastid DNA of Pcc is unique amongst the apicoplast DNAs of *Plasmodium* spp.

## Materials and Methods

### Parasite DNA

The purified genomic DNA of *P. chabaudi chabaudi* CB was a gift from Jean Langhorne group in the Division of Parasitology at MRC-NIMR, UK. Briefly, the DNA was obtained from parasite infected mice using a protocol reviewed and approved by the Ethical Review Panel of the MRC-NIMR and approved and licensed by the UK Home Office as governed by law under the Animals (Scientific Procedures) Act 1986 (Project license 80/1904). The animals were handled in strict accordance with the “Code of Practice Part 1 for the housing and care of animals (21/03/05)” available at http://www.homeoffice.gov.uk/science-research/animal-research/.

### High Throughput Sequencing (HTS)

Eight micrograms of total genomic DNA of *Plasmodium chabaudi chabaudi* isolate CB (Pcc CB) obtained from the blood of infected mice was fragmented in a microTUBE with an Adaptive Focused Acoustics fiber (Covaris, Woburn, Massachusetts) using an S2 focused ultra-sonicator (Covaris) set at 5% duty cycle; intensity 4; 200 cycles/burst for 90 s. In order to minimize the undesirable bias that affects the sequence analysis of extremely A/T-rich DNA such as that of *Plasmodium* spp. [8], we processed the fragmented DNA using a NEXTflex PCR-Free DNA Sequencing Kit (BIOO Scientific, Austin, Texas) and NEXTflex PCR-Free barcode 1 (BIOO Scientific) following the protocol provided with the kit. The processed DNA was applied to an agarose gel on E-Gel iBase (Life Technologies, Carlsbad, California) and fragments that ran as 400–500 bp were collected to form the sequence library for further analysis. After QC test and quantification, the library of DNA was analyzed on a HiSeq 2000 sequencer (Illumina, San Diego, California) and paired-end sequencing data of 100 nt/read were collected following the standard protocol. The raw data were deposited in the European Nucleotide Archive (ENA) under the accession number ERP002313.

HTS reads were aligned on reference sequences using CLC Genomic Workbench software package (CLC Bio, Aarhus, Denmark) either to obtain the consensus sequence and/or count the number of matching reads.

### PCR Direct Sequencing

To minimize errors accumulating during steps of molecular cloning, part of the nucleotide sequence of Pcc CB plastid DNA that we were unable to obtain simply by analyzing the HTS data was determined by PCR direct sequencing. Pcc CB plastid DNA containing the rRNA gene cluster was amplified from total genomic DNA by PCR with oligonucleotides 1095 (annealing to *trnH*; see Table 4) and 1096 (*sufB*) as below: 20 ng of Pcc CB total genomic DNA was added to 80 µL of reaction mixture containing x1 concentration of Reaction Buffer IV (Thermo Fisher Scientific, Waltham, Massachusetts), MgCl_2_ (2 mM), dNTP (0.4 mM each) and primers (250 nM each). The mixture was divided into two and each 40-microliter aliquot was dispensed into a 0.2 ml PCR tube. After adding 2 U of KAPA Taq DNA polymerase (KAPA Biosystems, Cape Town, South Africa), the PCR reaction was carried out in a thermal cycler with initial denaturation at 94°C for 2 min followed by 40 cycles of (denature at 94°C for 10 s, annealing at 53°C for 30 s, extension at 63°C for 270 s). To monitor the reaction, 1 µL was analyzed by electrophoresis on an agarose gel. To the remaining sample in each tube, 8 U of FastAP thermosensitive alkaline phosphatase (Thermo Fisher Scientific) and 80 U of NxGen Exonuclease I (Lucigen, Middleton, Wisconsin) were added, and each mixture was incubated at 37°C for 20 min, before both enzymes were inactivated at 80°C for 15 min. The nucleotide sequence of the PCR products was determined by Beckman Coulter Genomics (Essex, UK) using Sanger sequencing methodology and the oligonucleotides listed in the Table 4 as primers.

**Table 4:** Oligonucleotides used for PCR amplification and sequencing.

Name	Nucleotide sequence (5' >3')	Target a	Strand b
853	GATGAAGTCGTAACAAGGTA	*rrs*	sense
1005	TACTCTAGGGATAACAGGCT	*rrl*	sense
1095	GAACTCATATAAAAGAAACCAC	*trnH*	antisense
1096	CATTCAAAAAATGACCAATCTG	*sufB*	antisense

a Gene containing the annealing site.

b Of the target gene.

### Codon Usage Analysis

Codons present in all annotated protein CDS (total of 31) in the apicoplast genome were counted for the two Pcc isolates, AS (AB649423.1) [7] and CB (HF563595/HF563596) (this work), and compared with those of *P. falciparum* C10 (X95275.2/X95276.2) [6] for which decoding tRNA species have been proposed [10]. The *rpoC2* ( = *rpoD*) gene, in which a frame shift has been proposed [6], was regarded as two separate protein CDS (*rpoC2A* and *rpoC2B*).

### Estimation of Relative Copy Numbers

Relative copy number between each nuclear chromosome and the apicoplast was estimated from the average coverage of the entire length of each DNA by HTS reads in Table 2. Relative copy number for the trnR(ACG)*-trnM-1 gene cluster and the *trnR(ACG) -trnM-2* gene cluster (Fig. S1D) and between *trnT-1* and *trnT-2* (Fig. S1E) was estimated from the number of HTS reads containing characteristic variants. Because the reference sequences were short, each read was mapped on it as a single-end read. As the distance between variant sites 1 and 2/3 (Fig. S1D) was longer than 100 bp, there were no reads containing all three variant sites. However, analysis of paired end reads (Fig. S1C) suggested that those variants were exclusively linked together. Thus the count of each cluster was obtained as the sum the numbers of reads with variation at either site 1 or sites 2/3.

## Supporting Information

Figure S1 **High throughput sequence analysis of** ***Plasmodium chabaudi chabaudi*** **CB plastid DNA.** HTS reads of a library prepared from Pcc CB total genomic DNA were aligned on the plastid DNA sequence of Pcc isolate AS [7](A), *P. berghei* plastid DNA sequence containing *trnR(ACG)* [7](B) or Pcc AS plastid DNA sequence around *trnM* (C), the consensus of the two *trnR(ACG)-trnM* gene clusters in the Pcc CB plastid DNA (D) or Pcc AS *trnT* (E), using CLC Genomics Workbench (CLC BIO, Aarhus, Denmark). Reads that matched the reference sequence as a pair are shown in blue, whereas single matching reads are either in green (matching forward) or red (matching reverse). At the end of some reads where the sequence does not match the reference is shown in paler color, and internal residues that are different from those in the reference sequence are highlighted. Gaps are indicated by a space (A) or “–” (B–D). The position of each variation representing the type of gene cluster/gene is indicated on top of (D) and (E). Only reads with a significant high quality at the site of variation (highlighted) were counted, and the results are given in a table (inset in D and E).
(PDF)

Figure S2 **Hypothetical heterodimeric form.** Genes are color-coded as in Figure 2. Homologous recombination at any part of Pcc CB plastid DNA between one molecule each of forms A and B (Figure 2) will generate a unique heterodimeric form (Form C). Although such a form's presence has not been confirmed, it is possible that the Pcc plastid DNA occurs in this form at some stage of parasite development (see text). Note that this form has two units of a gene cluster (boxed) containing the same set of genes (except that *trnR(ACG)* has been degraded to trnR(ACG)*) in the same order as the IR unit of regular *Plasmodium* plastid DNA.
(PDF)
