# Correction: The Unique Structure of the Apicoplast Genome of the Rodent Malaria Parasite *Plasmodium chabaudi chabaudi*

**DOI:** 10.1371/annotation/f9f809fc-34b8-42c8-acf3-f8b2616a5f44

**Published:** 2013-11-07

**Authors:** Shigeharu Sato, Abdul K. Sesay, Anthony A. Holder

There were multiple errors in Table 1. Please see a corrected version of Table 1 here: http://www.plosone.org/corrections/pone.0061778.t001.cn.pdf

