# Supplementary figures and images for: The Unique Structure of the Apicoplast Genome of the Rodent Malaria Parasite Plasmodium chabaudi chabaudi
Source: PLoS One. 2013 Apr 16;8(4):e61778. doi: 10.1371/journal.pone.0061778 (PMC3627918; doi:10.1371/journal.pone.0061778)

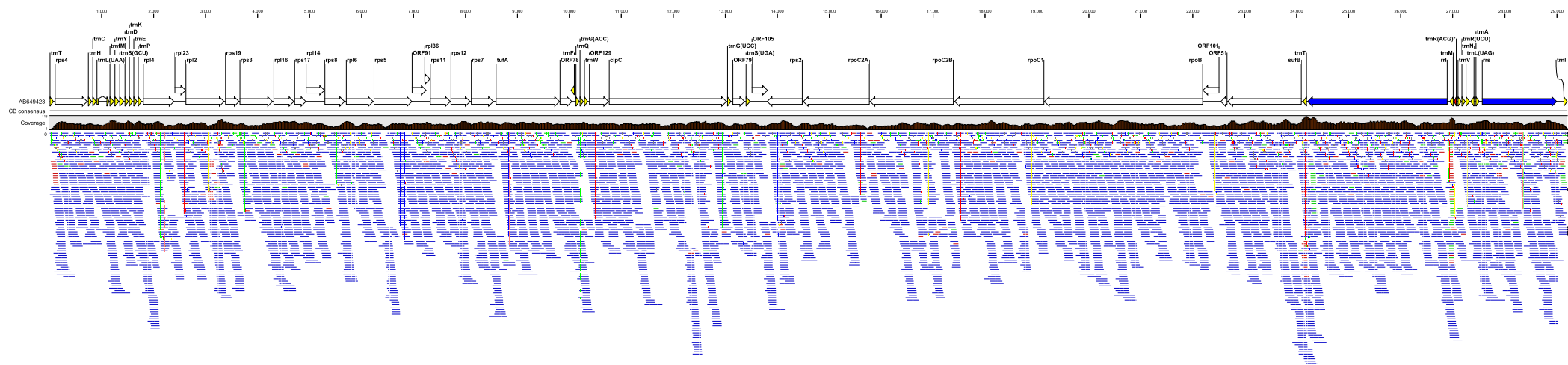





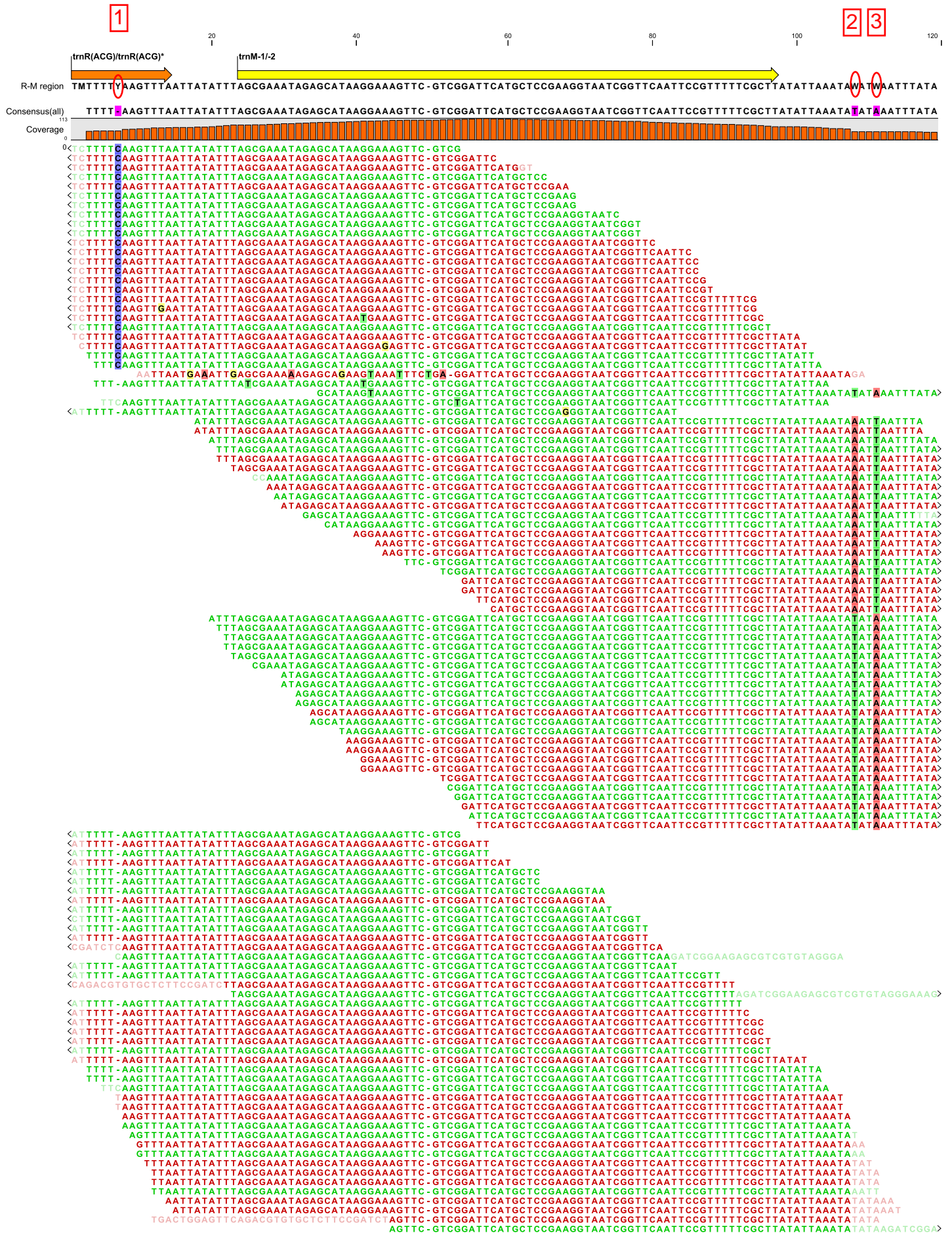

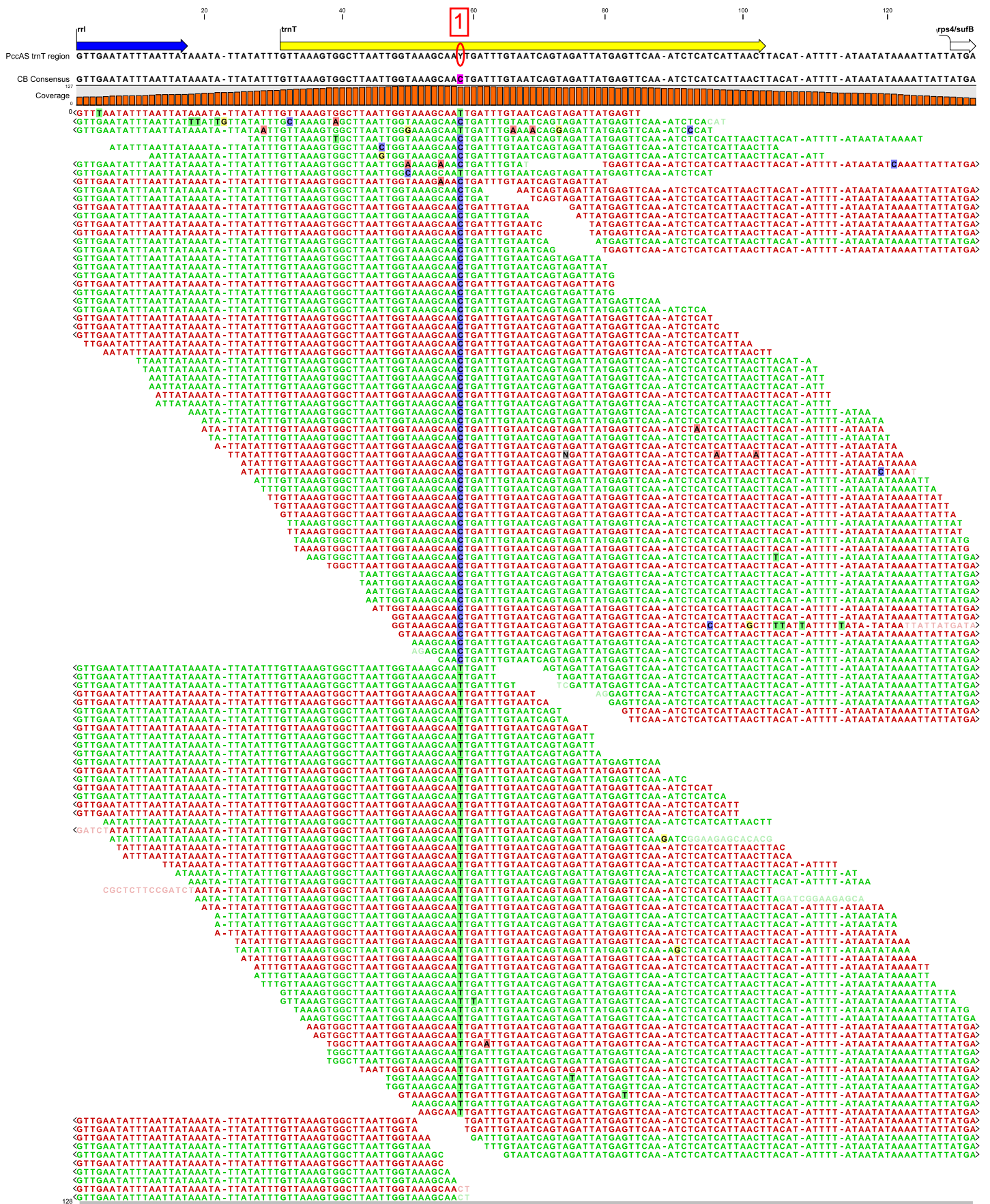

| Gene          | 1 | Reads | %     |
|---------------|---|-------|-------|
| <i>trnT-1</i> | C | 62    | 52.5  |
| <i>trnT-2</i> | T | 56    | 47.5  |
| Total         | Y | 118   | 100.0 |

Supplement: Figure S1 — High throughput sequence analysis of Plasmodium chabaudi chabaudi CB plastid DNA. HTS reads of a library prepared from Pcc CB total genomic DNA were aligned on the plastid DNA sequence of Pcc isolate AS [7](A), P. berghei plastid DNA sequence containing trnR(ACG) [7](B) or Pcc AS plastid DNA sequence around trnM (C), the consensus of the two trnR(ACG)-trnM gene clusters in the Pcc CB plastid DNA (D) or Pcc AS trnT (E), using CLC Genomics Workbench (CLC BIO, Aarhus, Denmark). Reads that matched the reference sequence as a pair are shown in blue, whereas single matching reads are either in green (matching forward) or red (matching reverse). At the end of some reads where the sequence does not match the reference is shown in paler color, and internal residues that are different from those in the reference sequence are highlighted. Gaps are indicated by a space (A) or “–” (B–D). The position of each variation representing the type of gene cluster/gene is indicated on top of (D) and (E). Only reads with a significant high quality at the site of variation (highlighted) were counted, and the results are given in a table (inset in D and E). (PDF) [file pone.0061778.s001.pdf]

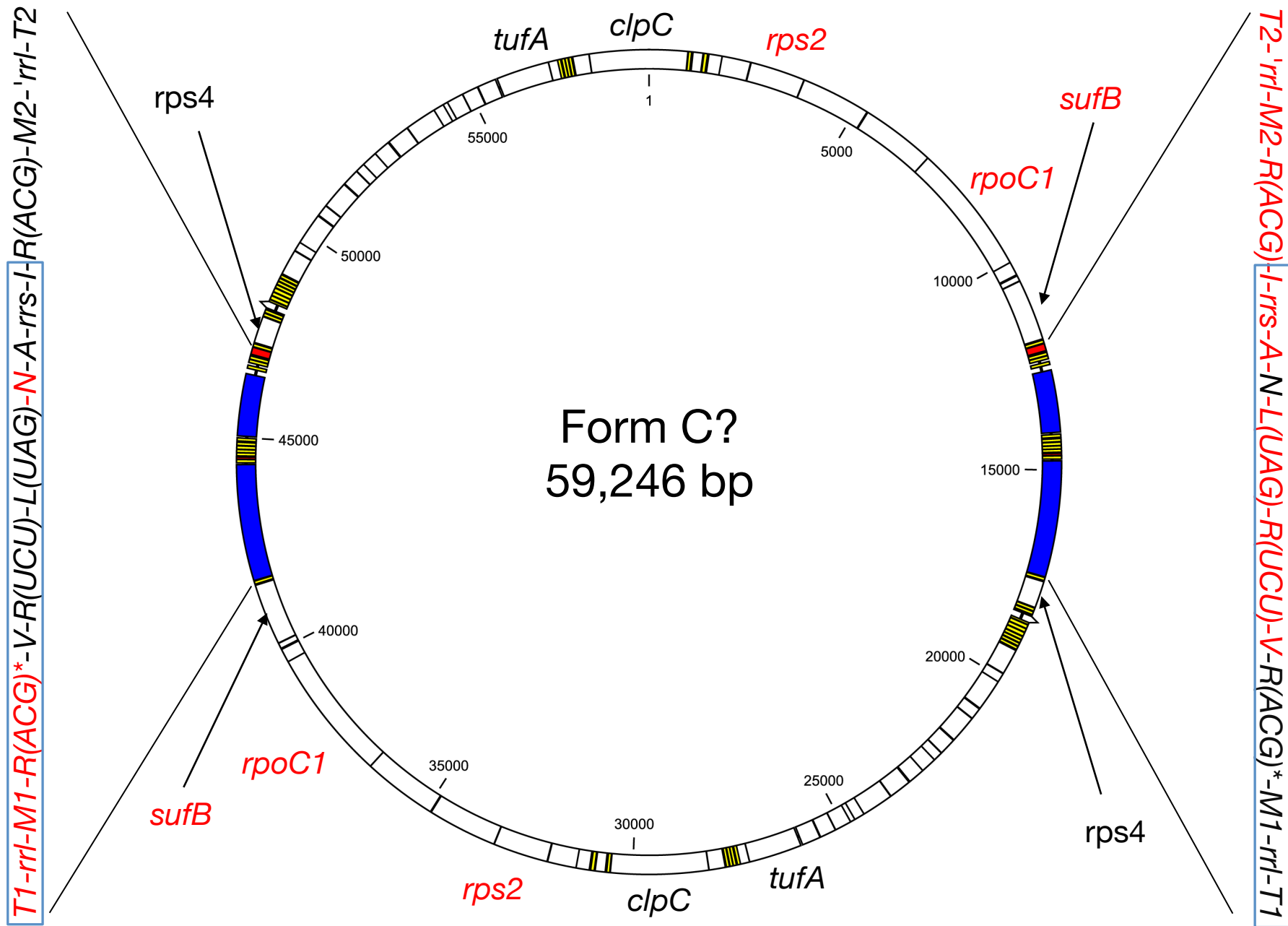

Supplement: Figure S2 — Hypothetical heterodimeric form. Genes are color-coded as in Figure 2. Homologous recombination at any part of Pcc CB plastid DNA between one molecule each of forms A and B (Figure 2) will generate a unique heterodimeric form (Form C). Although such a form's presence has not been confirmed, it is possible that the Pcc plastid DNA occurs in this form at some stage of parasite development (see text). Note that this form has two units of a gene cluster (boxed) containing the same set of genes (except that trnR(ACG) has been degraded to trnR(ACG)*) in the same order as the IR unit of regular Plasmodium plastid DNA. (PDF) [file pone.0061778.s002.pdf]
